# The role of socio-economic status in the decision making on diagnosis and treatment of oesophageal cancer in The Netherlands

**DOI:** 10.1038/sj.bjc.6603374

**Published:** 2006-10-10

**Authors:** E P M van Vliet, M J C Eijkemans, E W Steyerberg, E J Kuipers, H W Tilanus, A van der Gaast, P D Siersema

**Affiliations:** 1 Department of Gastroenterology and Hepatology, Erasmus MC – University Medical Center Rotterdam, PO Box 2040, 3000 CA Rotterdam, The Netherlands; 2 Department of Public Health, Erasmus MC – University Medical Center Rotterdam, PO Box 2040, 3000 CA Rotterdam, The Netherlands; 3 Department of Surgery, Erasmus MC – University Medical Center Rotterdam, PO Box 2040, 3000 CA Rotterdam, The Netherlands; 4 Department of Oncology, Erasmus MC – University Medical Center Rotterdam, PO Box 2040, 3000 CA Rotterdam, The Netherlands

**Keywords:** oesophagus, neoplasm, social class, histology, staging, therapy

## Abstract

In the United States (USA), a correlation has been demonstrated between socio-economic status (SES) of patients on the one hand, and tumour histology, stage of the disease and treatment modality of various cancer types on the other hand. It is unknown whether such correlations are also involved in patients with oesophageal cancer in The Netherlands. Between 1994 and 2003, 888 oesophageal cancer patients were included in a prospective database with findings on the diagnostic work-up and treatment of oesophageal cancer. Socio-economic status of patients was defined as the average net yearly income. Linear-by-linear association testing revealed that oesophageal adenocarcinoma was more frequently observed in patients with higher SES and squamous cell carcinoma in patients with lower SES (*P*=0.02). Multivariable logistic regression analysis showed no correlation between SES and staging procedures and preoperative TNM stage. The adjusted odds ratio (OR) for stent placement was 0.82 (95% CI 0.71–0.95), indicating that with an increase in SES by 1200 €, the likelihood that a stent was placed declined by 18%. Patients with a higher SES more frequently underwent resection or were treated with chemotherapy (OR: 1.15; 95% CI 1.01–1.32 and OR: 1.16; 95% CI 1.02–1.32, respectively). Socio-economic factors are involved in oesophageal cancer in The Netherlands, as patients with a higher SES are more likely to have an adenocarcinoma and patients with a lower SES a squamous cell carcinoma. Moreover, the correlations between SES and different treatment modalities suggest that both patient and doctor determinants contribute to the decision on the most optimal treatment modality in patients with oesophageal cancer.

Oesophageal carcinoma is currently on the sixth place of estimated cancer deaths worldwide (Parkin *et al*, 2001). Patients with oesophageal cancer have a dismal prognosis, as more than 50% of patients have already locally advanced carcinoma, lymph node metastases, or distant metastases at the time of presentation (Lightdale, 1999).

In the United States (USA), a correlation between socio-economic status (SES) of patients and the histology of oesophageal cancer has been demonstrated. Patients with a higher SES had a higher incidence of oesophageal adenocarcinoma, whereas squamous cell carcinoma was more frequently found in patients with a lower SES (Brown *et al*, 2001).

It has also been reported that patients with a lower SES were less likely to have localised cancer at diagnosis, compared with patients with a higher SES in the USA. This correlation was found for various cancers, that is, carcinomas of the breast (Li *et al*, 2003; Ward *et al*, 2004), uterine cervix (Ward *et al*, 2004) and oesophagus (Silverstein *et al*, 2002).

Finally, for breast cancer (Li *et al*, 2003; Ward *et al*, 2004) differences in the use of specific treatment modalities were present for SES in the USA. Patients with a higher SES were more likely to undergo a more appropriate treatment modality compared to patients with a lower SES, meaning that patients with a lower SES were at risk to receive unsatisfactory health care.

It is unknown whether in European countries a correlation is present between SES of patients with oesophageal cancer on the one hand, and tumour histology, staging approach, preoperative TNM stage and treatment modality on the other hand. In the present study we investigate these correlations in The Netherlands.

## PATIENTS AND METHODS

### Patients and database

In the Erasmus MC Rotterdam, The Netherlands, a prospective database is 2-weekly updated with information on patients who have been diagnosed with and treated for oesophageal or gastric cardia cancer from January 1994 until now. The database contains information on general characteristics, preoperative investigations and treatment modalities employed in these patients. In this study, we analysed findings from patients collected in the database between January 1994 and October 2003. In total, 1088 patients with oesophageal or gastric cardia cancer were seen in this period. Socio-economic status could be determined in 1078 patients. Of these, 888 patients had squamous cell carcinoma or adenocarcinoma of the oesophagus. In the remaining 190 patients, gastric cardia carcinoma (*n*=107) or oesophageal cancer with another histology (*n*=83) was found and these patients were excluded from the analysis. Information, not present in the database, but necessary for this study, was obtained from the electronic ‘hospital information system’, which contains additional clinical information on these patients. From the electronic ‘hospital information system’, we retrospectively collected information on whether a cancer located at the gastrooesophageal junction was in fact an oesophageal or a gastric cardia cancer. Other information retrospectively obtained from the electronic ‘hospital information system’ included information on chest X-rays (performed or not, and if yes the result), bronchoscopies (performed or not, and if yes the result) and CT scan results. Furthermore, the preoperative TNM stage was retrospectively determined using the results of the preoperative investigations performed.

### Socio-economic status

We defined SES of patients as the average net yearly income of income receivers with 52 weeks of income. The Central Office of Statistics in The Netherlands (CBS, The Netherlands) collects information on net yearly income, which is collected on a zip code level. Each zip code area represents approximately 4000 inhabitants. The database contains zip codes of patients and SES of patients was determined by means of these zip codes. Information on net yearly income was used from 1999. The use of information on SES with zip codes has been reported in other studies as well (Franks and Fiscella, 2002; Franks *et al*, 2003; Yoo and Thuluvath, 2004; Stern *et al*, 2005).

The SES varied between €11 800 and €22 100. For this study, we divided SES into three equal parts, that is, group 1: <€14 600 (*N*=295), group 2: €14 600–€15 800 (*N*=291) and group 3: >€15 800 (*N*=302). In The Netherlands, the mean net yearly income was 15 900 € in 2000 (Central office of statistics (CBS), January 2006).

### Tumour histology

Tumour histology was determined by means of investigation of biopsy specimens from the oesophageal tumour, which were obtained by endoscopy and examined by an experienced gastrointestinal pathologist. Patients were subdivided into those with oesophageal squamous cell carcinoma or adenocarcinoma. Oesophageal adenocarcinoma was present if the carcinoma was found in Barrett's oesophagus, or, in the absence of Barrett's oesophagus, if more than 50% of the adenocarcinoma was found in the oesophagus.

### Staging approach

Preoperative investigations, which are commonly performed in patients with oesophageal carcinoma, include chest X-ray (Stein *et al*, 2001), endoscopic ultrasonography (EUS) (Vickers and Alderson, 1998), CT scan (Maerz *et al*, 1993), ultrasound (US) of neck (Griffith *et al*, 2000) and abdomen (van Overhagen *et al*, 1992) and bronchoscopy (Riedel *et al*, 1998) in case of a carcinoma at or above the level of the carina. Patients who were included in this study were predominantly diagnosed in regional centers. After diagnosis, presumably curable patients are referred to our centre where they undergo (repeat) preoperative staging investigations. For all patients included in this study, only preoperative investigations performed in our centre were taken into consideration.

### Preoperative TNM stage

The preoperative TNM stage was determined as a result of the preoperative staging investigations. The T stage describes the depth of infiltration of the cancer into the different layers of the oesophageal wall. T stage was subdivided into T1 to T4, with a T1-carcinoma representing infiltration into the mucosa (T1m) or submucosa (T1sm), a T2 infiltrating into the muscularis propria, a T3 extending through the muscularis propria and a T4 infiltrating into surrounding organs or vessels. The N stage indicates the absence (N0) or presence (N1) of regional lymph node metastases and the M stage describes the absence (M0) or presence (M1) of distant metastases, with M1 stage being subdivided into an M1a- and M1b-stage (Fleming *et al*, 1997). When an item of the TNM-staging system was not available, this was staged as unknown. For example, if T stage was unknown, T stage was considered as Tx.

### Treatment modality

Treatment modalities, which were performed in the patients with oesophageal carcinoma, were an oesophageal resection, stent placement, chemotherapy, radiation therapy, a combination of chemotherapy and resection, or a combination of chemotherapy, radiation therapy and resection. For each patient, it was determined whether oesophageal resection, chemotherapy, radiation therapy or stent placement had been performed.

### Statistical analyses

Linear-by-linear association testing (*χ*^2^ testing) was used to determine a correlation between SES and tumour histology, extent of preoperative investigations, TNM stage and treatment modality.

For preoperative investigations, multivariable logistic regression was performed to correct for confounders. The included covariates were age, gender, tumour histology, comorbidity, tumour location and SES divided by 1200. The SES was divided by 1200 as the possible effect per euro was expected to be small. The number 1200 includes the difference between the lowest and highest income of group 2 (€ 14 600–€15 800). The number 1200 is, however, not a universal number used in multivariable logistic regression analysis. If the difference between the lowest and highest income of group 2 had been, for example, 1400, we would have divided SES by 1400. Comorbidity comprised all other disorders of patients that required medical treatment, such as cardiac or lung diseases. Tumour location was divided into five groups, that is, cervical, upper 1/3, middle 1/3 and lower 1/3 thoracic oesophagus and gastrooesophageal junction.

For N and M stages, we also performed multivariable logistic regression. M stage was subdivided into M0 and M1 stage, with M1 stage containing both M1a and M1b stages. The included covariates were age, gender, tumour histology, comorbidity, tumour location, tumour stage, preoperative investigations and SES divided by 1200. Multivariable logistic regression was not performed for T stage, as T stage was subdivided into four groups, that is, T1–T4, and for multivariable logistic regression the dependent variable should be dichotomous.

To correct for confounders in the possible correlation between SES and treatment modality, multivariable logistic regression was performed. We included the covariates age, gender, tumour histology, comorbidity, tumour location, tumour stage (TNM stage), preoperative investigations and SES divided by 1200.

Software used for analysis was SPSS (SPSS, Chicago, IL, USA). All *P*-values were based on two-sided tests of significance. A *P*-value <0.05 was considered as statistically significant.

## RESULTS

### Patient and tumour characteristics

Patient and tumour characteristics of the 888 patients with oesophageal carcinoma who were included in this study are shown in Table 1.

### Tumour histology

Table 2 shows the number of patients with squamous cell carcinoma or adenocarcinoma per income group. We found a lower percentage of oesophageal squamous cell carcinoma patients with increasing income. In contrast, the percentage of adenocarcinoma cases increased with higher SES (*P*=0.02).

### Staging approach

The numbers of patients who underwent EUS, CT scan, US neck, US abdomen, chest X-ray or bronchoscopy per income group are shown in Table 3. The linear-by-linear association test was only statistically significant for bronchoscopy (*P*=0.04), demonstrating that patients with a lower SES underwent more often a bronchoscopy. *P*-values for EUS (*P*=0.92), CT scan (*P*=0.14), US neck (*P*=0.44), US abdomen (*P*=0.34) and chest X-ray (*P*=0.48) were not statistically significant.

Table 4 shows the results of the multivariable logistic regression analyses. This table shows that the adjusted odds ratios (ORs) of the preoperative investigations were not statistically significant. The reason that bronchoscopy was not statistically significant in multivariable logistic regression analyses, while it was statistically significant in the linear-by-linear association test, was that bronchoscopy was more often performed in patients with squamous cell carcinoma compared to patients with adenocarcinoma (data not shown).

### Preoperative TNM stage

T, N and M stages per income group are shown in Table 5. T stage was unknown in 284 patients, and in these patients T stage was considered as Tx (Table 5). The linear-by-linear association test was not significant for T (*P*=0.97), N (*P*=0.68) and M stage (*P*=0.46).

In Table 6, the results of the multivariable logistic regression analyses are shown. It was found that the adjusted ORs of N (OR: 0.91; 95% CI 0.81–1.03) and M stage (OR: 0.93; 95% CI 0.81–1.07) were not statistically significant.

### Treatment modality

The numbers of patients who underwent resection, stent placement, chemotherapy or radiation therapy per income group are shown in Table 7. In 40 patients, no treatment was given. In the remaining 848 patients, more than one treatment modality has been employed in a subgroup of patients. The linear-by-linear association test was statistically significant for resection (*P*=0.001), showing that more resections were performed in patients with a higher SES. For stent placement, the linear-by-linear association test was also statistically significant (*P*=0.001). The negative correlation between SES and stent placement shows that fewer stent placements were performed in patients with a higher SES.

The results of the multivariable logistic regression analyses are shown in Table 8. It was found that the adjusted OR for stent placement was still statistically significant with a value of 0.82 (95% CI 0.71–0.95), meaning that with an increase in SES by 1200 €, the likelihood that a stent was placed declined by 18%. Furthermore, the adjusted ORs for resection and chemotherapy were also just statistically significant (OR: 1.15; 95% CI 1.01–1.31 and OR: 1.16; 95% CI 1.02–1.32, respectively), showing that resection and chemotherapy were more often performed with increasing SES. No correlation was found between SES and radiation therapy (OR: 1.04; 95% CI 0.90–1.22).

## DISCUSSION

In this study, a statistically significant correlation was demonstrated between SES, defined as average net yearly income of income receivers with 52 weeks of income, and histology of an oesophageal carcinoma. The incidence of squamous cell carcinoma declined and the incidence of adenocarcinoma increased with increasing SES. Our analyses demonstrated no correlation between SES and extent of staging procedures and between SES and preoperative TNM stage. A statistically significant negative correlation was however present between SES and stent placement, whereas a statistically significant positive correlation was present between SES and undergoing resection and between SES and undergoing chemotherapy. No correlation was found between SES and undergoing radiation therapy.

Well-known risk factors for oesophageal squamous cell carcinoma include smoking and alcohol consumption (Brown *et al*, 2001; Engel *et al*, 2003; Crew and Neugut, 2004). Gastrooesophageal reflux disease and obesity are identified risk factors for oesophageal adenocarcinoma (Engel *et al*, 2003; Crew and Neugut, 2004). In the USA, adenocarcinoma is more often found in patients with a higher SES, whereas squamous cell carcinoma is more common among patients with a lower SES (Brown *et al*, 2001). This previously observed correlation between SES and tumour histology was also found in the present study performed in The Netherlands. Although we had no information on risk factors in the patients with oesophageal cancer in this study, our results suggest that the higher prevalence of squamous cell cancer in the lower SES patients is due to more common smoking habits and alcohol consumption in these patients, whereas in patients with a higher income risk factors for GERD are more prominent. This is line with findings on smoking and alcohol consumption in the literature (Schnohr *et al*, 2004; Honjo *et al*, 2006).

In The Netherlands, general practitioners are the gatekeepers of the health care system, meaning that patients usually first consult the general practitioner for symptoms before being referred to a hospital (Kulu-Glasgow *et al*, 1998). In general, it is true that there is a low threshold and there are no economical barriers for patients to consult a general practitioner. In the present study, no statistically significant correlation was found between SES and performing preoperative staging investigations and between SES and TNM stage. This is likely to be explained by the fact that health insurance covers almost all people in The Netherlands, resulting in a similar access to health care faculties for all income groups.

In the USA, differences in the use of treatment modalities for oesophageal cancer have been reported for race. Non-Caucasian patients had a higher risk of receiving a less than optimal treatment compared with Caucasian patients, that is, non-Caucasian patients were less likely to receive an oesophagectomy and more likely to receive chemotherapy and/or radiation therapy (Dominitz *et al*, 2002). Factors that have been previously reported to be important in the differences in treatment modalities for both race and SES in the USA included differences in attitudes toward invasive procedures, disease severity, access to care (Dominitz *et al*, 2002), differences in undergoing staging procedures (Merrill *et al*, 2000), and issues related to health insurance (Mandelblatt *et al*, 1999).

The treatment preferences and attitudes toward invasive procedures of patients in the USA could be important explanations for the differences in use of treatment modalities in the USA. It is possible that treatment preferences and attitudes toward invasive procedures were also factors of importance in The Netherlands. In the present study, stent placement was more often performed in patients with a lower SES and oesophageal resection and the administration of chemotherapy were more common in patients with a higher SES. This might well suggest that patients in a higher income class are more eager to explore all therapeutic options, even experimental, to overcome the malignant disease they are suffering from. This is however speculative and we have no firm evidence to confirm this option. Furthermore, doctor contributions might also be important in the decision making on the most optimal treatment modality in patients, as it can be suspected that doctors are more willing to discuss all treatment options with patients if they are in the same income class, which often represents the same educational level.

In the USA, patients with a higher SES were more likely to have a localised cancer stage at diagnosis, compared with patients with a lower SES (Silverstein *et al*, 2002). Patient with locally advanced carcinoma or distant metastases will not receive an oesophageal resection and more often undergo treatment with chemotherapy and/or radiation therapy, which likely explains the differences in treatment modalities between different income classes in the USA. In the present study, no differences in preoperative TNM stage were found and as a consequence, disease stage is probably not important in the correlation between SES and treatment modality in The Netherlands.

In the USA, non-Caucasian patients were more often understaged, that is, underwent fewer preoperative staging investigations, in comparison with Caucasian patients (Merrill *et al*, 2000). In the present study, no correlation was found between SES and performing preoperative investigations and, as a consequence, this factor could not be an explanation for the correlations found between SES and treatment modality.

It has been reported that the health insurance status of a patient has an effect on the use of treatment modalities. For non-small-cell lung carcinoma, it has been shown that patients with private insurance were more likely to undergo a lung resection compared with patients without private insurance (Greenberg *et al*, 1988). In The Netherlands, almost all (>99%) inhabitants have a health insurance, resulting in similar health care services for all income groups. For that reason, differences in health insurance cannot explain the correlation between SES and treatment modality in The Netherlands.

What are other possible explanations for the observed differences on the role that SES plays in the diagnosis and treatment of oesophageal carcinoma patients between the USA and The Netherlands? First, almost all patients in this study were Caucasian. In the USA, the patients had different ethnic backgrounds and it has been demonstrated that differences in performing preoperative staging investigations, TNM stage and use of treatment modalities were not only present for SES but also for race (Klabunde *et al*, 1998; Bach *et al*, 1999; Merrill *et al*, 2000; Dominitz *et al*, 2002; Tomar *et al*, 2004; Ward *et al*, 2004; Steyerberg *et al*, 2005). As a consequence, race might be a more important factor compared to SES.

Second, people who are unemployed usually receive welfare in The Netherlands. As a consequence, the contrast between low- and high-income patients is probably smaller in The Netherlands in comparison with the USA. Therefore, the contrast between poor and rich was probably too small in this study to demonstrate the presence of differences in performing preoperative staging investigations and TNM stage.

Third, differences are present in health insurance and access to care. The majority of lower SES patients in The Netherlands have health insurance, which they receive from the Dutch National Health Service, whereas patients with higher SES pay health insurance themselves. As a consequence, almost all people in The Netherlands have health insurance, which means that access to care is equal, and similar health care services are available for all income groups. In the USA, not all patients have similar health care insurance and service, which could be responsible for the differences between the USA and The Netherlands.

There are several limitations to this study. First, in the present database with oesophageal cancer patients, no direct measures of SES were available. Nevertheless, the zip code of nearly all patients was present in the database. The Central Office of Statistics (CBS, The Netherlands) has designed a measure of SES by zip code representing the median net yearly income of an average of 4000 inhabitants. This information was used to determine the SES of individual patients. A disadvantage of this method is that an aggregate measure of SES was used for the SES of each individual patient. Another disadvantage could be that SES was relatively roughly determined, as it was estimated on the SES of an average of 4000 inhabitants, because there was no measure at the individual level available. Nevertheless, it has been demonstrated that health differences could be slightly more prominent when a more accurate measure of SES is used (Smits *et al*, 2005), suggesting that the differences could be underestimated in this study. In our opinion, it is unlikely that using a more accurate measure of SES would have changed the pattern of correlations.

Second, in the zip code area, the population is heterogeneous for economic characteristics, that is, not all persons in that particular zip code area will have the same SES. In the present study, the assumption was made that SES was homogeneous within the zip code area. As a consequence, all persons in one zip code area had an equal SES. Nevertheless, it could be possible that the SES of a patient was higher or lower than the average SES of the corresponding zip code area.

Third, in this study, only 888 patients were included who had oesophageal squamous cell carcinoma or adenocarcinoma. This is a relatively low number of patients to determine whether correlations existed between SES and characteristics of oesophageal cancer.

Fourth, the patients who were included in this study were a selection of all patients with oesophageal carcinoma in the southern part of The Netherlands. Usually, oesophageal carcinoma is diagnosed in regional centers, that is, centers with fewer than 10 patients per year. After diagnosis, patients often undergo preoperative staging investigations in these centers. Subsequently, they may be treated in the regional centre or, more often, are referred to our centre with a volume of more than 100 patients with oesophageal cancer per year (van Vliet *et al*, 2006). Patients in whom distant metastases were present according to the preoperative investigations performed in the regional centers were however only sporadically referred to our centre, which resulted in a relatively low number of patients with distant metastases in this study (Table 5). Furthermore, it is unknown whether other factors, such as SES or education level, played a role in the referring pattern of patients to our centre.

In conclusion, a significant correlation was found between SES of patients with oesophageal cancer and tumour histology. The negative correlation between SES and stent placement and the positive correlation between SES and resection and SES and chemotherapy suggest that patients in a higher income class more often do an utmost effort to overcome their disease. Furthermore, doctor contributions may be important in the decision making on treatment modality.

## Figures and Tables

**Table 1:** Patient and tumour characteristics of patients with oesophageal carcinoma (*n*=888)

**Characteristics**	
Mean age±s.d. (years)	62.7±10.1
	
*Gender* (%)	
Male	678 (76)
Female	210 (24)
	
*Histology of tumour* (%)	
Squamous cell carcinoma	388 (44)
Adenocarcinoma	500 (56)
	
*Location of tumour* (%)	
Cervical	10 (1)
Upper 1/3 thoracic	43 (5)
Middle 1/3 thoracic	158 (18)
Lower 1/3 thoracic	406 (46)
Gastrooesophageal junction	271 (30)

**Table 2:** Number of patients with oesophageal SCC or AC per income group

	**Socio-economic status in Euro**		
**Histology**	**<14 600 (*N*=295)**	**14 600–15 800 (*N*=291)**	**>15 800 (*N*=302)**
SCC (%)	147 (50)	119 (41)	122 (40)
AC (%)	148 (50)	172 (59)	180 (60)

Linear-by-linear association test: *P*=0.021.

AC, adenocarcinoma; SCC, squamous cell carcinoma.

**Table 3:** Numbers of patients with EUS, CT scan, ultrasound neck, ultrasound abdomen, chest X-ray or bronchoscopy per income group

	**Socio-economic status in Euro**			
**Investigation**	**<14 600 (*N*=295)**	**14 600–15 800 (*N*=291)**	**>15 800 (*N*=302)**	***P*-value^a^**
EUS (%)	199 (68)	203 (70)	205 (68)	0.915
CT scan (%)	175 (59)	169 (58)	161 (53)	0.138
Ultrasound neck (%)	263 (89)	253 (87)	263 (87)	0.444
Ultrasound abdomen (%)	188 (64)	178 (61)	181 (60)	0.341
Chest X-ray (%)	211 (72)	202 (69)	208 (69)	0.481
Bronchoscopy (%)	84 (29)	59 (20)	64 (21)	0.036

a Linear-by-linear association test.

CT, computed tomography; EUS, endoscopic ultrasonography.

**Table 4:** Multivariable logistic regression to determine whether a correlation existed between SES and preoperative investigations in patients with oesophageal carcinoma

**Investigation**	**OR**	**95% confidence interval**	***P*-value**
EUS	0.997	0.887–1.120	0.955
CT scan	0.960	0.861–1.071	0.466
Ultrasound neck	0.997	0.845–1.175	0.968
Ultrasound abdomen	0.957	0.858–1.068	0.434
Chest X-ray	0.968	0.862–1.088	0.590
Bronchoscopy	0.951	0.828–1.092	0.473

Covariates: age, gender, tumour histology, comorbidity, tumour location and SES/1200.

CT, computed tomography; EUS, endoscopic ultrasonography; OR, odds ratio; SES, socio-economic status.

**Table 5:** T, N and M stages per income group

	**Socio-economic status in Euro**			
**Stage**	**<14 600 (*N*=295)**	**14 600–15 800 (*N*=291)**	**>15 800 (*N*=302)**	***P*-value** *
*T stage*				0.972
T1 (%)	7 (2)	7 (2)	6 (2)	
T2 (%)	28 (10)	31 (11)	25 (8)	
T3 (%)	136 (46)	144 (49)	151 (50)	
T4 (%)	29 (10)	19 (7)	21 (7)	
Tx (%)	95 (32)	90 (31)	99 (33)	
				
*N stage*				0.680
N0 (%)	166 (56)	167 (57)	175 (58)	
N1 (%)	129 (44)	124 (43)	127 (42)	
				
*M stage*				0.459
M0 (%)	225 (76)	235 (81)	238 (79)	
M1a (%)	29 (10)	26 (9)	27 (9)	
M1b (%)	41 (14)	30 (10)	37 (12)	

* Linear-by-linear association test.

**Table 6:** Multivariable logistic regression to determine whether a correlation existed between SES and preoperative N and M stages in patients with oesophageal carcinoma

**Stage**	**OR**	**95% confidence interval**	***P*-value**
N	0.913	0.811–1.029	0.137
M	0.932	0.813–1.068	0.310

Covariates: age, gender, tumour histology, comorbidity, tumour location, tumour stage, preoperative investigations and SES/1200.

OR, odds ratio; SES, socio-economic status.

**Table 7:** Numbers of patients with oesophageal resection, stent placement, chemotherapy or radiation therapy

	**Socio-economic status in Euro**			
**Treatment**	**<14 600 (*N*=289)**	**14 600–15 800 (*N*=280)**	**>15 800 (*N*=291)**	***P*-value** *
Resection (%)	154 (52)	176 (61)	197 (65)	0.001
Stent placement (%)	86 (29)	61 (21)	55 (18)	0.001
Chemotherapy (%)	117 (40)	123 (42)	132 (44)	0.317
Radiation therapy (%)	43 (15)	56 (19)	47 (16)	0.753

* Linear-by-linear association test.

**Table 8:** Multivariable logistic regression to determine whether a correlation existed between SES and treatment modality in patients with oesophageal carcinoma

**Treatment**	**OR**	**95% confidence interval**	***P*-value**
Resection (%)	1.152	1.008–1.317	0.038
Stent placement (%)	0.822	0.712–0.949	0.008
Chemotherapy (%)	1.155	1.015–1.315	0.029
Radiation therapy (%)	1.043	0.895–1.215	0.592

Covariates: age, gender, tumour histology, comorbidity, tumour location, tumour stage, preoperative investigations and SES/1200.

OR, odds ratio; SES, socio-economic status.
